# New Drugs, Appliances, and Things Medical

**Published:** 1890-12-06

**Authors:** 


### NEW DRUGS, APPLIANCES, AND THINGS MEDICAL.

[All preparations, appliances, novelties, etc., of which a notice is desired, should be sent for The Editor, to care of The Manager, 140, Strand, London, W.C.]

### CORSETS OR NOT?

To wear or not to wear corsets: that is the question. Like most other questions which engage the male mind a little and the female mind a good deal, there is much to be said on both sides: much to be said and plenty of people to say it. Even doctors, who so notoriously agree with each other, disagree on this topic; whilst ladies who, it is well known, hate to take opposite sides on any question, nevertheless find themselves in different camps when the subject of corsets is "up." "Fools rush in where angels fear to tread," and we feel that we walk desperately near that precipice which separates folly from wisdom when we venture to say so much as a single word on the question. Nevertheless, what is a man without courage? It is better to march forward with heroes and philosophers into a twenty feet ditch, and thereby, like the famous Russian army, to make it safe for other people to walk across, than to seek a mean security and a reputation for wisdom by affecting the silence and the sightlessness of the sphinx. We give our vote for corsets, medical though our profession be. Let no high-nosed professional brother, or angry and double-skirted professional sister, point the finger of scorn at us; or, at least, not until they have heard our apologia, and our exposition of corsetical principles according to the standards of the Hospital.

Corsets claim our allegiance because they improve the human figure. This they certainly do if they are well made. By the human figure is not here meant the figure which nature makes and turns out, but the figure as it appears when milliners and dressmakers have spent their wits upon it. Our contention is that the properly corseted woman of Regent Street and the parks is as superior to the uncorseted as a well accoutred sergeant of the Forty-second Highlanders is to a raw recruit of the Puddleton Militia in his fatigue jacket. The human figure in its natural state is, of course, much more beautiful and graceful than any result which the milliner or the tailor can produce; but then we cannot have the human figure in its natural state on any terms: history, philosophy, Mrs. Grundy, Worth, and the milliners alike forbid it.

Seeing then that the human animal is to be clothed, we ought all to devote a reasonable share of intellect to clothing considerations. Corsets have these advantages, that they give a definite, and as we have now come to believe, a graceful shape to the figure; and they serve the necessary purpose of furnishing a suitable structure from which other clothing can be suspended. Now, the ordinary clothing of a woman must be suspended either from the shoulders or from the waist. Much of it, in fact, is suspended from the waist, and since that is so, it is infinitely better that it be suspended from stiffened corsets than from the upper part of the unprotected abdomen. There are two chief objections to stays: one is that they are worn too tight; the other, that they fit badly, and so make injurious pressure at particular spots. But those objections entirely disappear when the corsets are properly made and properly worn. Let these two conditions be fulfilled, and we have an article of dress which has the advantage of being both beautiful and useful.

The reason why the subject of corsets is now discussed in these pages is that Madame Wood, of 131, Edgware Road, some time ago sent us samples of abdominal belts and corsets. It was easy for a medical man, who is necessarily familiar with abdominal belts, to see whether or not they were well and skilfully made. As a matter of fact they were, or they would not have been sent to us. But the case was quite different with the corsets. Even here, too, the doctor could see that they were well made, and so adapted to the outlines of the figure as to make equal and uniform pressure over all the parts. But of what avail was all this mechanical perfection unless the corsets fulfilled the one indispensable condition of pleasing the wearer? Resolved not to speak with authority on a subject which he had not explored to its depths, the writer took a heroic resolution, and asked his wife to order a pair of Madame Wood's corsets and to have them made in the highest style of perfection. This resolution was equivalent to the outlay of about thirty shillings, and was therefore very virtuous on the part of a husband who had not been asked to spend such a considerable extra sum. The result, however, was admirable. The wearer is charmed with Madame Wood's corsets, and declares that she has not had any before that have been either so comfortable or so elegant, even though they may have cost nearly twice the money. Now this is not a puff, lady readers, for the writer does not know Madame Wood from Adam, or rather from Eve. But since he had the samples sent to him, and felt that the subject was of some importance to all sorts and conditions of women, he made up his mind, even at the risk of being misunderstood, to investigate it with reasonable thoroughness, and to write of it with straightforwardness and simplicity. We do not care one atom where ladies get their corsets made, but we do advise them to wear only such as fit both easily and accurately. These two conditions being fulfilled, women may regard with perfect equanimity all the highly seasoned news paper stories of dreadful deaths from tight lacing.

### SPECTACLE APPLIANCES.

Messrs. J. Raphael and Co., 13, Oxford Street, London, have recently patented a very simple but useful device for making spectacles more comfortable to wear. Everybody who has worn spectacles knows how difficult it is to get a pair which are neither so tight as to pinch when adjusted behind the ears, nor so loose as to become a nuisance rather than an aid to sight. The new patent consists of very small half spheres of indiarubber which can readily be adjusted to the arms of the spectacles, and so produce comfort and security without trouble of any kind. Raphael's indiarubber spheres are well worth a trial.

